# Action researchers as “orchestrators” of co-innovation: a theoretical and methodological framework

**DOI:** 10.1186/s12913-024-10779-6

**Published:** 2024-04-09

**Authors:** Marianne Eliassen, Cathrine Arntzen, Lina Forslund, Morten Nikolaisen, Patrik Alexandersson, Astrid Gramstad, Andreas Hellström

**Affiliations:** 1 Department of Health and Care Sciences, UiT The Arctic University of Norway, PO Box 6050 Langnes, Tromsø, 9037 Norway; 2 Centre for Healthcare Improvement (CHI), Chalmers University of Technology, Gothenburg, 412 96 Sweden; 3 Division of rehabilitation Services, University Hospital of North Norway, Tromsø, 9037 Norway

**Keywords:** Co-innovation, Health care services innovation, Action research, Democratic validity

## Abstract

**Background:**

With the increasing complexity of health care services, more comprehensive and integrated services need to be designed. Action researchers are encouraged to facilitate multiactor participation and user-centered approaches to initiate service development. However, “orchestrating” co-innovation, in which actors have diverse attitudes, agendas, positions of power, and horizons of understanding, is challenging, and a framework that supports action researchers in co-innovation studies lack. The purpose of this article was to explore how action researchers can facilitate multiactor engagement and handle possible challenges and stimulate creativity among diverse stakeholders.

**Methods:**

We have studied and discussed two Scandinavian cases of rehabilitation innovation (for cancer patients and persons with acquired brain injury) where two research teams with action research approaches have acted in an orchestrating role to create co-innovation.

**Results:**

We identified four themes that are essential for action researchers to facilitate collaborative and creative co-innovation processes: (1) relational power reflexibility, (2) resource integration, (3) joint understanding, and (4) the facilitation of creativity. These mutually dependent themes constitute a theoretical and methodological framework for of co-innovation.

**Conclusions:**

This paper offers a contribution that supports action researchers in orchestrating diverse actors and their contributions in co-innovation processes.

## Background

The complexity of health care services across a wide range of disciplines is rapidly increasing [1]. Due to multimorbidity and interacting sociocultural influences, health care institutions are among the most complex and interdependent entities in society [2–5]. Historically, health care organizations have been structured and designed within a society where infections and other delimited issues were predominant, and the specialization of services was a prerequisite for delivering adequate services. Public bureaucrats have instinctively sought to deconstruct problems into smaller, more manageable parts in an attempt to “tame” them. However, organizational problems are deemed “wicked and unruly” necessitating solutions that embrace rather than oversimplify complexity [6].

With these issues in mind, there has been a rapidly increasing interest in designing comprehensive and integrated services to meet the challenges of complex health care needs [4, 7, 8]. The complex issues that exist in and across organizations and sectors cannot be addressed by any single discipline, as they should be acknowledged as pluralistic and complex, and approached by a broad range of actors.

Designing new services that address the challenges of today’s complex and fragmented health care services call for innovative solutions that consider the wide perspectives of the multiple stakeholders involved. In this study, we build on the definition of co-innovation by Saragih and Tan [9]: “Co-innovation is defined as a shared work of generating innovative and exceptional design conduct by various actors”. What constitutes an “exceptional design” varies in each unique context. However, in this study, we fucus on innovations created by multiple actors who represent diverse rules, attitudes, agendas, knowledges, and institutional cultures. Within this framework, co-innovations are innovations transcend existing sectorial, organizational, or professional boundaries (both tangible and intangible) to create new ways of delivering services that generate public value. In accordance with Lee, Olson, and Trimi [10], co-innovation represent a new innovation paradigm where various actors engage to generate shared values. This paradigm of co-innovation can assist organizations in creating shared value through convergence, collaboration, and co-creation, involving collaboration through open network to a greater extent than the previous paradigms of ‘closed-innovation,’ ’collaborative innovation,’ and ‘open innovation’ strategies [2, 10–12].

Researchers [13, 14] call for collaborative knowledge generation in the development of services that can meet the needs of integration and collaboration in health care services. This new research paradigm suggests a transformative role of service research, in addition to new ways of managing practice development, as academics work alongside other stakeholders to create public value [13]. The collaborative knowledge generation is believed to increase research impact by bridging the knowing-doing gap between the academic and practice fields [13, 14].

Action research (AR) has been advocated to be a suitable approach for researchers who wish to engage in innovation [15]. AR offers an alternative orientation to knowledge creation compared to a traditional notion of ‘evidence creation’ as a pre-existing truth about the world waiting to be discovered by researchers. Instead, action researchers aim to alter power dynamics, facilitating the empowerment of stakeholders, including patients and their families, in collaborative knowledge creation [15, 16]. A fundamental assumption in such approaches is that scientific knowledge is socially constructed through collective reflections and that the planning, execution, dissemination, and implementation of research are not separate actions but are deeply interconnected and contextual. Within such an understanding, the relationship between researchers and research users must be interpreted as a co-constructive partnership.

In AR, researchers are not solely tasked with evaluating effects; they are also deeply involved and accountable for the processes of knowledge construction in the development and design of services. Eriksson and Hellström [17] argue that AR can strengthen public services and systems, not only by producing knowledge about existing services but also by integrating researchers’ knowledge and skills in the design and innovation of services. This often requires action researchers to manage the complexity of orchestrating a diverse group of actors, in line with Greenhalgh et al. [13], who call for researchers to address the processes of innovation in addition to the outcomes. In their literature review, Greenhalgh et al. [13] summarized findings on various forms of co-creation, which they defined as collaborative knowledge generation between researchers and stakeholders from the practice field. In their research, they found that the literature tends to fucus on particular programs and projects, whereas less attention has been paid to the processes of the research, and strategies that inform and shape how we define and judge research. Osborn et al. [18] highlight that co-innovations are not a normative good, as they also have the potential to lead to ‘co-destruction’. Additionally, they claim that theories and research tend to focus on the role of service users in co-innovations, confusing co-innovations with user-led services, while the role of other actors, such as service professionals, are less studied [18]. There is a need for increased knowledge about the processes of co-innovation, which may form a framework that support action researchers in their value creation.

Co-innovation of health care services involves a wide range of stakeholders, such as patients, families, clinical professionals, administrators, and the wider community represented by volunteers and nonprofit organizations. It has been argued that this multiple-actor approach provides complementary value-in-context and contextualized experiences that are relevant for sustainable service designs [6, 11], as well as enhancing creative problem-solving and innovation [19, 20]. A meta-analysis by Damanpour [21] confirms that a high degree of diversity among the involved actors has a positive impact on innovation in public organizations.

However, multi-actor involvement also brings challenges, as different stakeholders often have different organizational and professional cultures, diverse attitudes, positions of power, and horizons of understanding, which can be obstacles to collaboration [19, 22]. Donetto et al. [23] state that a simplification of democratization can increase oppression and social exclusion of the already oppressed and therefore call for increased attention to critically explore how power relations among participants can affect collaborative processes. Researchers engaging in co-innovation must be able to operate outside the traditional narrow framework of “evidence creation”, as their role includes integrating varied perspectives and contributions of diverse stakeholders and facilitating creativity, shared experiences, and value creation [6]. In this paper, we refer to action researchers’ new role of facilitating innovation among diverse actors as “orchestrators” of co-innovation.

Co-innovation studies in health care settings often describe the roles of other actors, such as social enterprises [24], health care professionals [25], and public managers [6], social entrepreneurs or third sector organizations [20], while the role of action researchers is only sparsely described [14]. Although the paradigm of co-innovation calls for researchers to engage in organizational development, democratic processes [14], creative thinking and shared knowledge generation [13], there is a lack of descriptions on how to perform such actions. Strategies for overcoming barriers to collaboration, such as different views, conflicts of interest and power discrepancies, are needed.

The purpose of this article is to explore the methodology of co-innovation, specifically how action researchers can facilitate multi-actor engagement and stimulate creativity among diverse stakeholders. This paper has evolved through thorough discussions among action researchers, based on experiences from two large co-innovation projects within the field of rehabilitation research. In this paper, we aim to discuss overarching themes that can constitute a methodological framework for orchestration of co-innovation.

First, we will present two empirical cases of co-innovation processes from health care settings in Sweden and Norway. Next, we describe researchers’ strategies of orchestration through experiences from the empirical cases. Finally, we discuss how these strategies may serve as a methodological framework for researchers’ orchestration of co-innovation and discuss how this may contribute to increasing the quality of AR in the public sector.

## Methods

The discussions that we present in this study are obtained through action researchers’ abductive reasoning [26] and overarching discussions based on large action research projects aiming for co-innovation in health care services. The authors of this paper, engaged as action researchers in the two co-innovation cases that we will describe in the following.

### Study context

Two independent empirical cases of co-innovation-based AR from health care settings in Sweden and Norway are presented to illustrate and discuss potential strategies in co-innovation processes.

The Nordic welfare states have available and extensive public services that are mainly publicly organized and provided, and health care financing is mainly composed of tax revenues [27]; these are among the most service-intensive states in the Western world [28]. The principle of equal rights is one of the most central principles of the Nordic welfare state, and publicly funded services should be offered to all citizens regardless of their financial situation, social status, gender, or age [28]. It has been argued that the traditional public management strategies of specialization, differentiation, segmentation and decentralization create fragmented welfare services, such that collaborative and integrated services are hampered [17, 29]. Collaborative efforts to redesign services to be more integrated are emphasized in the Nordic context. Despite some differences, such as the consumption of services and the degree of decentralized services, the contexts of Norwegian and Swedish health care services are comparably similar.

The two cases that are presented in this article represent services that target persons with acquired brain injury (ABI) and cancer survivors and their significant others. Common to these user groups is that they often struggle with everyday activities due to reduced motor, psychosocial, and cognitive functioning. These issues are often neglected in a mono-professional, fragmented, and diagnosis-based health care system, and patients often experience a lack of continuity and coordination in service provision [30]. Both innovation projects target people with chronic or long-term conditions, with a focus on rehabilitation and health promotion. This means that both cases also involve many more actors than those limited to the health care sector.

Central to both co-innovation cases was the creation of services that could support patients and their families in physical, social, emotional, and cognitive challenges in everyday life. The initiatives of both cases were based on intersubjectivity and a life event or everyday life perspective, moving away from a medical discourse toward a matter of community integration [31]. Knowledge creation and continuous reflexive development [14] were central visions in both projects. These perspectives entailed a need for service innovations at the intersection between existing services, including welfare initiatives beyond typical health care services. Therefore, a co-innovation approach was applied in both cases.

### RehabLos, a rehabilitation model for persons with acquired brain injury (ABI) in Northern Norway

ABI can be caused by a variety of incidents, such as stroke, bacterial infections, neurosurgical operations, or trauma. This may lead to a wide range of impairments, such as decreased motor, cognitive, social, and emotional abilities, all of which are preconditions for functional everyday living. Consequently, people suffering from ABI often have complex and diverse rehabilitation needs, which involve not only the patient but also their significant others and, in a broader view, society as a whole [32]. Contemporary health care policies emphasize shorter hospital stays, and patients who do not have substantial impairments related to motor function or speech run the risk of being discharged to home without proper assessment or adequate follow-up [33].

Health service researchers (including five of this paper’s authors) at UiT, The Arctic University of Norway initiated RehabLos, a co-innovation project with the goal of developing a collaborative rehabilitation model to support community integration of people with mild or moderate ABI. The initiating researchers were all health care professionals (physiotherapists, occupational therapists, and nurses) who had experience with neurorehabilitation from clinical work, research, and education. Aiming for a more comprehensive rehabilitation model that targets community integration, the research group invited a wide range of stakeholders to participate in the project. The stakeholders included persons with ABI, significant others, multiprofessional health care staff from hospitals and primary care, coordinated care services, and representatives from the Labor and Welfare Administration. In total, 30 stakeholders participated in the project. The action researchers facilitated and designed the process, which included sending out invitations to attendees, arranging activities, and collecting data for academic purposes.

During a period of 12 months (April 2021-March 2022), the researchers initiated field work, two digital seminars and three all-day workshops aiming to co-innovate a cross sectorial rehabilitation model for people with ABI. The events included focus group discussions, individual interviews, service design activities, and plenary discussions. All the events were either video or audiotaped, and field notes were taken throughout the entire process.

### ‘Kraftens Hus’, enhancing psychosocial support for cancer patients and their families in Sweden

A cancer diagnosis can affect a person in multiple ways: physically, psychologically, socially, and existentially. Rehabilitation and better collaboration between authorities and welfare actors are needed to support cancer patients in returning to work and functional life. Cancer rehabilitation in Sweden is inadequate in many parts of the country, leaving patients with insufficient psychosocial support and struggling to find a new identity. Both patients and their relatives may need societal support in various ways, and multiple actors may be involved. At a societal level, there is an increasing realization of the need for strengthened collaboration between societal actors to create a better, more needs-adapted, more equitable, and legally secure welfare system.

The initiative for Kraftens Hus (The House of Power) came from the Patient and Relative Council at the Regional Cancer Center West (RCC West), which tested new ways for patients and relatives to contribute to the development of cancer care. A multiprofessional project group was created to facilitate the innovation process. The group consisted of a patient representative with personal experience living with chronic cancer, relatives, and actors from the local hospital, primary care, the municipality, the Social Insurance Agency, regional politicians, the Employment Service, local businesses, and civil society. In addition, an experienced action researcher and a service designer (two of the authors of this paper) experienced in innovations in various industries also participated.

To view the entire complexity from the perspective of those affected, not just their disease, the work was guided by a life event perspective [34], emphasizing a persons’ life events, rather than focusing on the medical disease when developing services. The project aimed to identify shortcomings and opportunities in organizational gaps, stressing the view that the main challenges lie in the “gap between” existing services.

During a period of seven months (May 2016-November 2016), seven co-innovation workshop, including focus groups and design activities (see detailed descriptions elsewhere [35]), were conducted with the aim of developing a service offer that supports the psychosocial needs of cancer patients. The co-innovation events were documented by field notes.

### Data generation and analysis

Data were generated throughout the processes of both the co-innovation case and are described thoroughly elsewhere. For detailed descriptions of the RehabLos project, see Forslund et al. [36] and Ellingsen et al. [37], and for the Kraftens Hus project, see Huzzard et al. [22] and Hellström [35].

Field notes and transcribed audio and video material from workshops and co-innovation meetings constituted the data for the analysis. Seven researchers (the authors of this paper) from both co-innovation projects arranged joint analysis workshops (two all-day physical workshops, and three digital meetings), where we analyzed data through a qualitative thematic approach [38]. In the first analysis workshop, the researchers presented summaries of fieldnotes, focus groups transcripts, and evaluation notes from both projects for each other to generate a common insight, and to enable transverse analysis that was representative for both co-innovation cases. We were interested in generating knowledge about the orchestration of the multiple contributions from different stakeholders. Therefore, we initially focused on the action researchers’ actions (both intended and spontaneous) that facilitated reactions among the participants. Patterns of similarities across the two cases gave rise to some preliminary themes of how action researchers orchestrate co-innovation. In the second analysis workshop, we focused on the participants activities and evaluations to map out facilitators and barriers for interactions and innovation. These facilitators and barriers were connected to the preliminary themes, which were refined in accordance with the new insights. During the following digital meetings, the researchers discussed and negotiated commonalities and discrepancies within the data. Through iterative discussions, moving between data and theory of co-innovation in an abductive manner [26], four common themes were eventually created. All the seven authors participated in the analyses, which generated a triangulation of multiple perspectives.

## Results

Based on experiences from the two Scandinavian cases, we identified four themes that were perceived as central for the action researchers’ orchestration of the co-innovation processes: (1) relational power reflexivity, (2) resource integration, (3) joint understanding, and (4) the facilitation of creativity.

### Relational power reflexivity

The first theme revolves around aspects concerning the diverse positions of power that the multiple stakeholders possess, and how action researchers should exercise reflexivity regarding this aspect when orchestrating co-innovation. We present strategies that were observed within the co-innovation cases, and the researchers’ rationales for these strategies. By reflecting on the diverse positions of power, the action researchers were able to facilitate activities that could level out the asymmetric relationships between diverse group of stakeholders.

Experiences from the Norwegian case of co-innovation for persons with ABI (RehabLos) demonstrate that the action researchers strategically arranged activities for leveling out asymmetrical power relations. While assembling a diverse group of stakeholders for innovation workshops, the importance of selecting a “neutral” venue was emphasized to ensure that all participants would feel equally included. The action researchers therefore booked a local conference facility for the arrangement of the three workshops. Initially, it was emphasized that all participants got to know each other. Everyone was provided with a name tag, and all group activities were initiated with a presentation of the respective stakeholders. The rationale for this was to build confidence and trust among the participants.

The researchers in RehabLos designed several activities that were carried out during the workshops to facilitate discussions and dialog among the various actors. During the first workshop, all actors were divided into focus groups to identify and discuss the needs of persons with ABI and challenges in current health care services. Although multi-actor engagement was perceived as essential for the co-innovation process, the researchers decided to arrange an initial homogenous group exclusively for users and significant others. This choice was made to create an arena where users and significant others could feel empowered by peer support and be able to bring forward their voices without interruptions from other stakeholders who could possibly hold a superior power position. In this group meeting, users and significant others were presented with the questions and themes for the multi-actor groups so that they were prepared and felt confident about engaging in the collaboration with other stakeholders in subsequent groups. Evaluations of the project revealed that this was valued by the users, who felt supported by their peers. Enhancing empowerment among the user group was intended to bring out the voices of the users and to bring forward “what really matters” in the meetings between all stakeholders. One of the care providers evaluated this to be motivating by stating: *“I really appreciated the fact that the users contribute to pinpoint what really is important! [I] experienced new enthusiasm and motivation”.*

In the Swedish Kraftens Hus case, relational power was also claimed to be an essential component. Before the initiative of Kraftens Hus was initiated, a group of patients had started a movement with the ambition to raise awareness about the difficult situation in which many cancer patients found themselves while trying to manage their lives. The impact was minimal, and a clear lesson was that they did not have access to or a mandate in the forums to drive this issue. With the support of the multiprofessional project group and RCC West, a broad platform was created, paving the way to drive the innovation project of Kraftens Hus. In this manner, the Swedish cancer project was placing the ownership of the project with the patient group. The rationale behind this, was to empower the users, leveling out a possible asymmetric power balance between service providers and service users. Through workshops and dialogs based on “the whole system in the room” (see e.g. [22, 39, 40]), the action researcher and the project team tried to establish an environment to encourage an open-minded approach, allowing for collective questioning of the existing order, rethinking of beliefs about what is possible, and redefining of solutions and approaches to meet the needs of individuals affected by cancer.

Another consideration made in the early phase of the RehabLos case was to reflect on the stakeholders’ previous relationships and how it could influence the group dynamics and the co-innovation processes. Some of the users, significant others, and professionals already knew each other. On the one hand, the established relationships were discussed as contributing to establishing confidence and trust. On the other hand, the action researchers tried to be sensitive to how the previous power relations influenced the innovation process, particularly in terms of who was to speak out and who remained silent. By moderating the group dialogs, the researchers facilitated joint engagement by all the participants.

Power inequity was not only discussed as a question of the power relations between users and “the others” but also a matter of relationships among all the involved stakeholders. In both cases, the researchers were aware that power and agency were central in this type of co-innovation, and there was a clear aim to reduce, or compensate for, power and relational diversities during the innovation processes. In both cases, the user groups were actors that might struggle to have their voice heard in society, with a weak impact against authorities and health care providers. As the researchers were the ones who facilitated all workshops, they were in position to mediate the different perspectives and facilitate a balanced power relation among the varied actors.

### Resource integration

The second theme displays how resource integration was perceived as a central element to succeed with co-innovation, and how it was important for action researchers to facilitate engagement by all stakeholders and visualize how diverse forms of resources could be integrated to complement each other. One participant claimed that succeeding with a complex service innovation was utopian unless one ensured resource contributions from all participants. In the project Kraftens Hus, the involvement of multiple stakeholders was deemed essential to ensure its effectiveness. The idea was that when the multiple actors saw the benefits of the activities, this could create a potential for a socially and economically sustainable service model. To achieve this, the participants were encouraged to engage in collaborative discussions to develop a common understanding of the value creation in the project. Some of the participants claimed that it was impossible for one single organization to arrange complex services for cancer patients alone, as the financial conditions were limited, and collaboration with contributions from multiple actors was perceived as a prerequisite to succeed. However, it was also recognized that resources were not limited to financial resources, as materials, knowledge, and information were also perceived as important contributions.

Recognizing that resources imply more than merely financial resources, the researchers in RehabLos worked strategically to integrate the more intangible resources in the form of experiences, perspectives, and competences. Participants were therefore strategically selected, to ensure resource contributions from a variety of stakeholders, including patients, significant others, organizations, sectors, and professions. To ensure that different perspectives and fields of competences were brought up, all stakeholders were divided into heterogeneous focus groups consisting of researchers, persons with ABI, significant others, and stakeholders from various parts of the welfare services. The action researchers assumed a role as mediators to ensure engagement by all participants. After one of the innovation sessions, several of the participants stated that they felt that their voices were heard, and one of the user representatives with ABI stated: *“I have felt like an equal partner. Of course, I know that there will always be someone who has preconceived opinions about brain damage’s self-awareness and reflective abilities. However, in this setting, I have met skilled professionals with good empathetic manners.”*

Anchoring the projects at an administration level was also perceived as important to ensure resource contributions from the diverse stakeholders. Therefore, the researchers in the RehabLos project initiated meetings with leaders from all the represented organizations in the project, and signed agreements were ensured before any activities in the project were conducted.

### Joint understanding

The third theme that was identified in this study was *joint understanding*. This theme arose from the observations of activities that action researchers performed to ensure that the diverse stakeholders were able to share a joint understanding of service challenges, user needs, and the service goals. Creating a joint understanding was perceived as a prerequisite for the stakeholders to ‘pull in the same direction’, leading towards co-innovation.

**Fig. 1:**
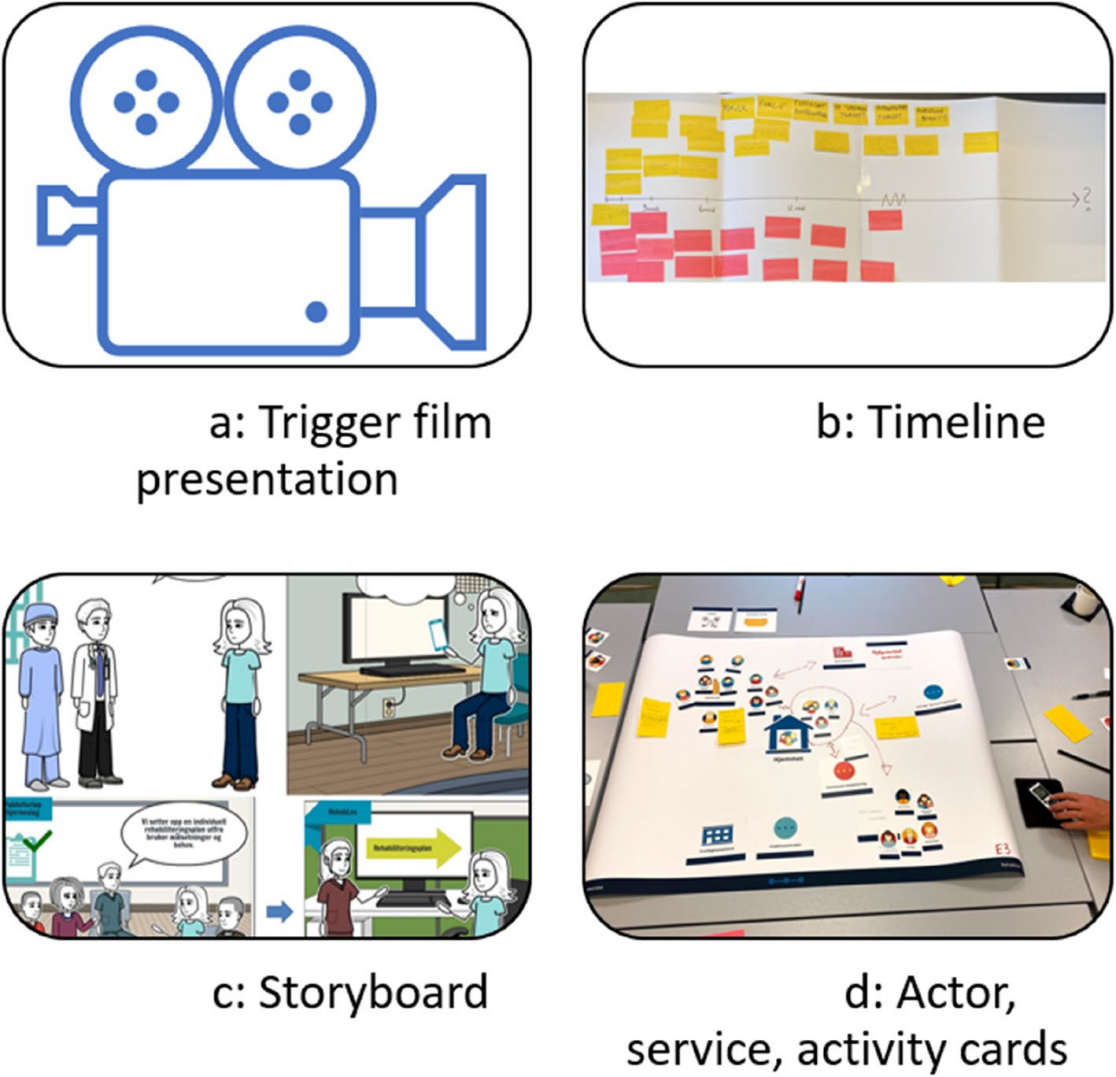
**a**-**d** Design activities to promote common engagement and creativity in a multi-actor co-innovation process

 In the RehabLos case, the researchers included “trigger films” as a strategy to create a common foundation for the involved actors. The researchers videotaped interviews with two persons with ABI and three health care providers to generate trigger films of patients and service providers who provided experiences with ABI and health care services. In the first innovation workshop, the trigger films were presented to all participants to create a common understanding and joint engagement in the user experiences (Fig. 1a).

Furthermore, a perspective that emphasized everyday life events, rather than a disease-oriented approach focusing on biomedical aspects, was chosen as an overall concept for the discussions and activities in the workshops to underpin user experiences. The researchers facilitated the focus group discussions by displaying a timeline that represented a user’s rehabilitation process after the occurrence of ABI (Fig. 1b). The rationale behind the user-focused timeline was to create a joint focus on the conduct of everyday life with which everyone could identify, thus preventing a conversation about medical diagnosis and organizational structures that potentially would exclude some of the actors from the dialog. As the cases included multiple stakeholders aiming to create innovation, it was emphasized to facilitate a ‘everyday life event-language’ where everyone perceived a shared understanding of the concepts and terminology used in the process. The rationale behind this was that a common language could contribute to better communication and understanding between stakeholders, and by that contribute to reducing barriers between stakeholders and thus increasing collaboration and cooperation. Building on an everyday life perspective was evaluated as beneficial, as it was described to represent the cornerstone of challenges for both ABI patients and their significant others and facilitated a “common language”. After the workshop, one participant stated: *“Participation in this project has contributed to increased insight and knowledge about the user group and challenges that they experience. I believe this will support me in my daily practice, consequently, increasing the quality of the services I provide.”* This perspective was therefore considered appropriate for tracking challenges in existing services, while simultaneously facilitating conversations and discussions characterized by ‘everyday language’ in which all stakeholders could engage and to which they could relate.

The researchers also presented a storyboard based on user experiences that represented a user’s meeting with current health care services (Fig. 1c). The rationale behind this was to bring to the forefront the voices of the users and to achieve a joint understanding of the potential challenges of a fragmented and complex health care system.

Visual tools were also used in the case of Kraftens Hus. For example, a four-minute film “What if” was used to visualize the shared vision and show how many different parties in society can be affected by a cancer diagnosis. The film represented the perspective of the person with cancer but also included how health care employees, the Swedish Social Insurance Agency, and people in the cancer patient’s everyday life are affected. The film had a clear message about shared responsibility and was therefore a natural choice for the action researchers to clarify the shared responsibility.

### The facilitation of creativity

The last theme that was identified in this study displays how action researchers facilitate creativity as a means for co-innovation. The rationale behind this strategy was that collective creativity was perceived to challenge the traditional mindsets of the participants and involved that the actors had to face their potential “taken-for-granted” views, accepting new ways of thinking, and embrace the perspectives of others. After the first workshop in the RehabLos case, one of the participants paradoxically evaluated that she felt that the participants were too unified and that she missed some constructive conflicts and contrasting views on the discussed themes. A possible explanation for this may have been that the action researchers had overlooked the promising potential of contradicting views, while they had strived for an equal horizon of understanding and an equalized balance of power.

This perspective gave rise to a new strategy for the researchers, who, in the second workshop, arranged for activities that intended to disturb preconceived understandings. The participants were provided with ‘actor cards’, ‘service cards’ and ‘activity cards’ (Fig. 1d) that represented actors, services and activities that were identified in the first workshop as relevant for the community integration of people with ABI and their significant others. The participants were challenged to discuss how different actors and services could be put together in new, creative ways of working and collaborating to meet the users’ needs. Different solutions were promoted and discussed. In evaluations, the participants supported the assumption that such visual tools facilitated creative thinking. Visualizing new ways of organizing services enabled the actors to see past traditional standards and organizational boundaries that are often taken for granted and possibly limit service provision. One of the participants stated that discussing possible solutions for new service design normally would be limited by questions about financial or organizational limitations. However, she stated, the innovation strategy of using visual tools enhanced creative discussions without bringing up limitations and constraints. These examples of how to use visual tools provide a strategy for action researchers to facilitate creative thinking that extends beyond the existing organizational frameworks.

## Discussion

In this section we will discuss how the four mutually dependent themes identified in this paper, can be utilized as a methodological framework for action researchers who orchestrate co-innovation processes. Second, we will discuss how this framework may contribute to increasing the validity of AR in the public sector.

### A framework for action researchers’ orchestration of co-innovation

Traditionally, health care services have been developed from a service-provider perspective, hence, roles and power relations between providers and users have been clearly demarcated, challenging substantial democratic processes [23]. However, the cases represented in this study, emphasize user experiences as a cornerstone in innovation, in line with descriptions of Osborne [12]. *The first theme* identified in this study, relational power reflexivity, shows that trust-building activities were important to facilitate joint engagement by the diverse actors, in line with Crosby et al. [6] who argue that collaborating partners build trust by sharing information and knowledge and by demonstrating competency and good intentions.

Exploring the position of the involved users and significant others was deemed essential in this study. The two cases had different strategies to support the empowerment of the users. While the ABI-project arranged peer-support meetings to build trust and empowerment among users to prepare for co-innovation activities, the cancer-project placed the project ownership with the user group and a non-profit organization. Nonprofit organizations are often trusted advocates that “speak on behalf of the interests of citizens”, and the majority are volunteers who are motivated by a desire to make a difference and to positively impact society [22]. This makes the third sector actors suitable as initiators for co-innovation projects, as they can facilitate trustful engagement by the users and significant others and in that matter contribute to empowering the users’ voice and neutralizing potential asymmetric power relations between the involved stakeholders. Projects that are initiated and “owned” by user representatives are in a unique position to bring forward the users’ voice and designing user-centered processes. However, one should also bear in mind that this means that the user group holds a hegemonic position compared to other actors, which may limit the perspectives of those with professional and organizational competence.

Based on these experiences, we put forward a proposition that empowering users can be facilitated by strategies that emphasize trust-building and peer-support activities, or strategies that provide the user group with project ownership. In addition, the imbalance of power between actors from varied facilities was discussed to possibly challenge the co-innovation. Action researchers should therefore reflect on the diverse power relations of all the involved stakeholders in the planning of co-innovation activities.

*The second identified theme*, resource integration, calls for action researchers to be sensitive to the diverse forms of resources varied actors may contribute with. It has been argued that resource contributions from multiple actors can support public services to better address complex issues [17]. In contributing to the enhanced quality of public services and generating public value, resource integration can serve as a frame that commits the involved actors. In co-innovation, resources must be understood as both tangible and intangible [17] as they may include experiences, contextual knowledge, professional knowledge, analytical competence, and creative engagement, in addition to material resources. Researchers’ contribution of facilitation and orchestration is therefore no exception. In the ABI case, the research team consisted of researchers with health care backgrounds, and in that matter, they had essential knowledge about the field of study. However, this position could again counter the neutral position of being an outsider. The positionality and structure of the relationship between researchers and practitioners may provide both benefits and limitations of a co-innovation process and should therefore be discussed in each individual case.

Based on these experiences, we propose that action researchers should be reflexive about how mediating activities and dialogs can facilitate engagement and contribution by all stakeholders. The researchers’ contribution, regarding their “neutrality” and knowledge to the field of practice, should also be acknowledged.

*The third theme* that we constituted in this study, was ‘joint understanding’, as action researchers in the two cases emphasized activities that could account for the diverse perspectives and understanding of the stakeholders. Differentiated organizational and professional cultures, horizons of understanding [19, 22], and even a certain way of speaking (“tribal language”) [41, 42] may challenge co-innovation. Crosby and Bryson [43] emphasize the importance of creating an early agreement about the nature of the problem when diverse actors collaborate for innovation. Additionally, one should strive for a dialog that does not involve “tribal language”, which will exclude certain participants.

To create joint understanding, both the presented cases in this study utilized an everyday life event-perspective, moving the focus away from medical disease and diagnostic terms. Additionally, the action researchers used visual tools such as “trigger films”, timelines, and actor cards as a response to situations where they identified a need for cross boundary interactions. Trigger films are suggested by Donetto et al. [23] and Windrum et al. [17] to create engagement among participants and facilitate a joint understanding of the varied actors’ roles. By using varied visual tools, the researchers aimed to facilitate dialog and reflections with a ‘common language’ while visualizing the complexity of health care systems. In this matter, visual tools functioned as boundary objects [44], connecting the multiactor perspectives through common “rules of the game”. Boundary objects are described as objects that may have different meanings in different social contexts but are nevertheless structured in a way that is common enough to be recognizable from varied perspectives and therefore are key objects in developing coherence across intersecting social worlds [44, 45]. Torfing [19] suggests that narratives, heuristic models, artifacts, and prototypes may function as boundary objects, and thereby facilitate collaboration across boundaries. In the co-innovation cases in this study, the action researchers chose visual tools for two intentions; first, to achieve power balance between the involved actors, and to facilitate activities of creativity. This is in line with how Kimble et al. [46] describe how strategically chosen artifacts can become boundary objects when managed particularly to enhance cross boundary interaction.

Based on experiences from both the co-innovation cases, we put forward a proposition that an everyday life event-perspective may facilitate a common ground for collaboration and propose that action researchers utilize visual tools as boundary objects when aiming to engage diverse stakeholders with different backgrounds and horizons of understanding.

*The fourth theme* elaborates on how action researchers facilitate creativity as a means for co-innovation. Based on feedback from participants, the action researchers in the ABI-project arranged activities that aimed for ‘constructive conflicts.’ This harmonizes with Crosby et al. [6] who argue that it is advantageous to create an appropriate disturbance of the collaborative process to encourage the participants to creative thinking. Crosby et al. [6] state that leaders who manage collaborative innovation processes need to facilitate participants to think outside the box and experiment in the face of imperfection rather than giving in to rule-following, risk avoidance, and safe retreat.

Innovation is often defined as the development and practical realization of new and creative solutions that challenge hegemonic views [47]. By applying creativity-generating methods, as shown in the examples in the presented cases, one may create unexpected associations with problems, which is also observed by Wegener [48].

Based on this, we propose that action researchers who orchestrate co-innovation should intervene with the intention to create situations in which the actors move out of their comfort zone to generate new ideas, identities, resources, and desires.

### Enhancing action research rigor in co-innovation

Co-innovation entails the inherent tension between collaboration and innovation, representing a paradoxical challenge. While collaboration thrives in the presence of a certain similarity in terms of background, education, and values between actors, innovation flourishes when different experiences, views and ideas complement and disturb each other, as it facilitates creative problem solving. Despite the strong promising opportunities of co-innovation, it follows that widely divergent views, ideas and interests may hamper a joint understanding, resulting in a ‘dialog of the deaf’ [49]. The results in this study display how action researchers involved in co-innovation can contribute to facilitate and orchestrate diverse actors and their contributions by leveling out the asymmetrical distribution of power, ensuring the integration of relevant resources, creating a joint understanding, and facilitating creativity.

We argue that these mutually dependent (and to a certain point overlapping) themes collectively constitute a unifying theoretical and methodological framework for orchestrating co-innovation that aims for public value. Such a framework may prepare action researchers who assume an orchestrating role in co-innovation and thus contribute to solving complex problems and creating public value. This interactive and highly involved role may also contribute to revitalizing the view of researchers, supporting a contemporary paradigm of knowledge creation as socially constructed and minimizing the gap between researchers and research users.

Herr and Anderson [50] have put forward the concepts of *dialogic validity, process validity, and democratic validity*, which refers to how quality and rigor of AR must be evaluated through other criteria than validity measures in traditional research. Dialogic validity refers to inclusion of varied actors with a wide range of perspectives [50]. Process validity refer to the researcher-practitioner relationships, in which trustful relations are expected to enhance innovation, problem solving, and learning [50]. Democratic validity refers to the equal terms of participation for all actors to ensure their varied experiences and insights from diverse contexts emerge [50]. The four themes identified in this study, can be interpreted as an operationalization of this set of validity criteria for AR. Hence, we argue that the results of this study can be utilized to increase the quality of AR in co-innovation processes.

### Study limitations

As the purpose of this article was to explore the action researchers’ orchestration of co-innovation, we have reported on overall discussions from two large co-innovation projects in Scandinavia. These studies were not initiated and designed to generate knowledge about action researchers’ role in co-innovation, as they aimed to innovate health care services. Therefore, the data that constitute the foundation for our results are merely a combination of data from the two cases, and reflections and discussion between the involved researchers. Therefore, rather than describing all data generating activities of the two cases in depth, we have briefly described the most relevant aspects of the cases, which are relevant to provide knowledge about the orchestration of co-innovation. Omitting some aspects of the projects means that there may be other relevant aspects that are not treated in this article.

Reporting on effects of AR is challenging as such practices are highly multifaceted and complex. Although we cannot conclude if the two cases that we present in this article have resulted in more effective services (which would require other service designs), experiences of the co-innovation process may provide valuable insights about the co-innovation processes that can be utilized for further development of the field of AR. A further exploration of the effects of the co-innovation strategies that are provided here, is needed.

## Conclusion

This work has been focused on exploring the methodology of co-innovation, as we have described how action researchers can orchestrate varied actors in a collaborative process with the goal of creating public value. By providing examples from two large studies in Scandinavia that engaged patients and other stakeholders in co-innovation, we have shown how action researchers can orchestrate co-innovation through redistribution of power, integration of resources, facilitation of joint understanding and creativity.

We have also discussed how these themes may support quality in AR, as they intervene with the concepts of dialogic, process, and democratic validity.

## Data Availability

As this article is a theoretical article based on examples from two projects, no distinct data are available publicly. However, it is possible to contact the authors for further details about the studies: marianne.eliassen@uit.no; cathrine.arntzen@uit.no; andreas.hellstrom@chalmers.se.

## Abbreviations

ABI: Acquired brain injury
AR: Action research
NSD: The Norwegian Social Data Services
RCC West: Regional Cancer Center West

## References

[1] Plsek PE, Greenhalgh T (2001). The challenge of complexity in health care. BMJ.

[2] Chandra Y, Shang L, Roy MJ (2022). Understanding healthcare social enterprises: a new public governance perspective. J Soc Policy.

[3] Glouberman S, Mintzberg H (2001). Managing the care of health and the cure of disease—part I: differentiation. Health Care Manag Rev.

[4] Greenhalgh T, Papoutsi C (2018). Studying complexity in health services research: desperately seeking an overdue paradigm shift. BMC Med.

[5] Kodner DL, Spreeuwenberg C (2002). Integrated care: meaning, logic, applications, and implications–A discussion paper. Int J Integr Care.

[6] Crosby BC, Hart ‘T, Torfing P (2017). Public value creation through collaborative innovation. Public Manag Rev.

[7] Plsek PE, Wilson T (2001). Complexity, leadership, and management in healthcare organisations. BMJ.

[8] Roberts JP, Fisher TR, Trowbridge MJ, Bent C (2016). A design thinking framework for healthcare management and innovation. Healthcare.

[9] Saragih HS, Tan JD (2018). Co-innovation: a review and conceptual framework. Int J Bus Innov Res.

[10] Lee SM, Olson DL, Trimi S (2012). Co-innovation: convergenomics, collaboration, and co‐creation for organizational values. Manag Decis.

[11] Elg M, Engström J, Witell L, Poksinska B (2012). Co-creation and learning in health‐care service development. J Serv Manag.

[12] Osborne SP (2018). From public service-dominant logic to public service logic: are public service organizations capable of co-production and value co-creation?. Public Manag Rev.

[13] Greenhalgh T, Jackson C, Shaw S, Janamian T (2016). Achieving research impact through co-creation in community‐based health services: literature review and case study. Milbank Q.

[14] Elg M, Gremyr I, Halldorsson Á, Wallo A (2020). Service action research: review and guidelines. J Serv Mark.

[15] Lifvergren S, Huzzard T, Hellström A (2015). Action research and healthcare. Act Res.

[16] Koch T, Kralik D. Participatory action research in health care. Singapore: Wiley; 2009.

[17] Eriksson E, Hellström A (2021). Multi-actor resource integration: a service approach in public management. Br J Manag.

[18] Osborne SP, Radnor Z, Strokosch K (2016). Co-production and the co-creation of value in public services: a suitable case for treatment?. Public Manage Rev.

[19] Torfing J (2019). Collaborative innovation in the public sector: the argument. Public Manag Rev.

[20] Windrum P, Schartinger D, Rubalcaba L, Gallouj F, Toivonen M (2016). The co-creation of multi-agent social innovations: a bridge between service and social innovation research. Eur J Innov Manag.

[21] Damanpour F (1991). Organizational innovation: a meta-analysis of effects of determinants and moderators. Acad Manag J.

[22] Huzzard T, Hellström A, Lifvergren S (2018). Whole system in the room: toward systems integration in healthcare. Health Commun.

[23] Donetto S, Pierri P, Tsianakas V, Robert G (2015). Experience-based co-design and healthcare improvement: realizing participatory design in the public sector. Des J..

[24] Chandra Y, Shang L, Roy MJ. Understanding healthcare social enterprises: a new public governance perspective. J Soc Policy. 2022;51.4: 834–55.

[25] Grenier C (2011). Structuring an integrated care system: interpreted through the enacted diversity of the actors involved—the case of a French healthcare network. Int J Integr care.

[26] Blaikie N (2007). Approaches to social enquiry: advancing knowledge.

[27] Rostgaard T (2012). Quality reforms in Danish home care–balancing between standardisation and individualisation. Health Soc Care Community.

[28] Vike H (2017). Politics and bureaucracy in the Norwegian welfare state: an anthropological approach.

[29] Valentijn PP, Schepman SM, Opheij W, Bruijnzeels MA (2013). Understanding integrated care: a comprehensive conceptual framework based on the integrative functions of primary care. Int J Integr Care.

[30] Sjetne IS, Holmboe O. Pasienters erfaringer med norske sykehus i 2019. Metodebeskrivelse og analyser for landet samlet [Patients’ experiences with Norwegian hospitals in 2019. Methods description and analyzes for the country as a whole]. In: *PasOpp-rapport 2020* Folkehelseinstituttet; 2020. https://www.fhi.no/contentassets/1ed1cf501b0a43d58415dcbb3ab889e6/metodebeskrivelse-og-analyser-for-landet-samlet.pdf

[31] Shaikh NM, Kersten P, Siegert RJ, Theadom A (2019). Developing a comprehensive framework of community integration for people with acquired brain injury: a conceptual analysis. Disabil Rehabil.

[32] Arntzen C, Moe S, Aadal L, Pallesen H (2019). Facilitating learning and change in the daily lives of stroke survivors: a comparative analysis of municipal stroke rehabilitation services in Norway and Denmark. Cogent Med.

[33] Bettger JP, McCoy L, Smith EE, Fonarow GC, Schwamm LH, Peterson ED (2015). Contemporary trends and predictors of postacute service use and routine discharge home after stroke. J Am Heart Assoc.

[34] Fransson M, Quist J. Livshändelser för gemensamma medborgarmöten i svensk förvaltning: Ett diskussionsunderlag. In: Nationella rådet för innovation och kvalitet i offentlig verksamhet. 2011.

[35] Hellström A, Holmlid S, Wetter-Edman K (2021). Design i tjanstesystem |Designing service systems|. Tjanstedesign Principer och praktiker [Service design Principles and practices] edn.

[36] Forslund L, Arntzen C, Nikolaisen M, Gramstad A, Eliassen M. Physiotherapy as Part of Collaborative and Person-Centered Rehabilitation Services: The Social Systems Constraining an Innovative Practice. Physiother Theory Pract. 2023:1–16. 10.1080/09593985.2023.2255893. 10.1080/09593985.2023.2255893 37676077

[37] Ellingsen G, Arntzen C, Forslund L, Nikolaisen M, Eliassen M, Gramstad A, Manskow U (2023). Designing digital systems for rehabilitation practices. Tidsskrift Omsorgsforskning.

[38] Braun V, Clarke V (2006). Using thematic analysis in psychology. Qualitative Res Psychol.

[39] Cooperrider DL, Stavros JM, Whitney D. The appreciative inquiry handbook: for leaders of change. Berrett-Koehler; 2008.

[40] Gustavsen B (1996). Action research, democratic dialogue, and the issue of critical mass in change. Qual Inq.

[41] Foronda C, MacWilliams B, McArthur E (2016). Interprofessional communication in healthcare: an integrative review. Nurse Educ Pract.

[42] Marion ADC, Pereira LC, Pinho DLM. The effect of interprofessional simulation practice on collaborative learning: a randomized controlled trial. J Interprof Care. 2023:1–8. 10.1080/13561820.2022.2147153. 10.1080/13561820.2022.2147153 36606366

[43] Crosby BC, Bryson JM (2010). Integrative leadership and the creation and maintenance of cross-sector collaborations. Leadersh Q.

[44] Star SL, Griesemer JR (1989). Institutional ecology,translations’ and boundary objects: amateurs and professionals in Berkeley’s Museum of Vertebrate Zoology, 1907-39. Soc Stud Sci.

[45] Akkerman SF, Bakker A (2011). Boundary crossing and boundary objects. Rev Educ Res.

[46] Kimble C, Grenier C, Goglio-Primard K (2010). Innovation and knowledge sharing across professional boundaries: political interplay between boundary objects and brokers. Int J Inf Manag.

[47] Osborne SP, Brown L (2011). Innovation, public policy and public services delivery in the UK. The word that would be king?. Public Adm.

[48] Wegener C (2020). Improvisation: Innovation-Fra nyhed til nærvær. Samskaping: Sosial innovasjon for helse og velferd. edn.

[49] Koppenjan JFM, Koppenjan J, Klijn EH (2004). Managing uncertainties in networks: a network approach to problem solving and decision making.

[50] Herr K, Anderson GL. The action research dissertation: A guide for students and faculty. London: Sage publications; 2014.

[51] WMA declaration of Helsinki. – Ethical principles for medical research involving human subjects. https://www.wma.net/policies-post/wma-declaration-of-helsinki-ethical-principles-for-medical-research-involving-human-subjects/. Accessed 11 June 2023. 19886379

